# Integrated Process for Sequential Extraction of Bioactive Phenolic Compounds and Proteins from Mill and Field Olive Leaves and Effects on the Lignocellulosic Profile

**DOI:** 10.3390/foods8110531

**Published:** 2019-10-29

**Authors:** María del Mar Contreras, Antonio Lama-Muñoz, José Manuel Gutiérrez-Pérez, Francisco Espínola, Manuel Moya, Inmaculada Romero, Eulogio Castro

**Affiliations:** 1 Department of Chemical, Environmental and Materials Engineering, University of Jaén, Campus Las Lagunillas, 23071 Jaén, Spain; alama@ujaen.es (A.L.-M.); jmgp0014@red.ujaen.es (J.M.G.-P.); fespino@ujaen.es (F.E.); mmoya@ujaen.es (M.M.); iromero@ujaen.es (I.R.); ecastro@ujaen.es (E.C.); 2 Center for Advanced Studies in Energy and Environment, University of Jaén, Campus Las Lagunillas, 23071 Jaén, Spain

**Keywords:** bioactive compounds, biorefinery, oleuropein, olive leaves, phenolic compounds, vegetable protein, ultrasound

## Abstract

The extraction of bioactive compounds in a biorefinery context could be a way to valorize agri-food byproducts, but there is a remaining part that also requires attention. Therefore, in this work the integrated extraction of phenolic compounds, including the bioactive oleuropein, and proteins from olive mill leaves was addressed following three schemes, including the use of ultrasound. This affected the total phenolic content (4475.5–6166.9 mg gallic acid equivalents/100 g), oleuropein content (675.3–1790.0 mg/100 g), and antioxidant activity (18,234.3–25,459.0 µmol trolox equivalents/100 g). No effect was observed on either the protein recovery or the content of sugars and lignin in the extraction residues. Concerning the recovery of proteins, three operational parameters were evaluated by response surface methodology. The optimum (63.1%) was achieved using NaOH 0.7 M at 100 °C for 240 min. Then, the selected scheme was applied to olive leaves from the field, observing differences in the content of some of the studied components. It also changed the lignocellulosic profile of the extraction residues of both leaf types, which were enriched in cellulose. Overall, these results could be useful to diversify the valorization chain in the olive sector.

## 1. Introduction

Olive tree cultivation is growing worldwide; the total area harvested was 10.8 million ha in 2017, which is three million more than in 1997 [1]. Thus, in addition to the main product, olive oil, high amounts of byproducts are generated. In particular, olive leaves (≈20%–25% by weight) are firstly generated during the tree pruning process and, secondly, in the mill leaves and thin branches (olive mill leaves) (≈4%–10% by weight) are separated together from olives using a blower machine [2,3,4,5]. This means that, for example, a hectare of olives trees could generate around 300–750 kg of olive leaves and 250 kg of olive mill leaves [3,4,5,6], or even more. These proportions may vary depending on the tree age, growing conditions, crop production, pruning intensity, local pruning practices, etc. [5,6]. Despite these large quantities, their industrial applications are still limited. In the worst scenario, these byproducts are burnt [6] and thereby contributing towards the emission of greenhouse gases.

Alternatively, olive leafy byproducts can be potential natural resources for obtaining valuable phytochemicals. Among them, oleuropein has revealed pharmacological potential in itself and as a starting material to develop new bioactive compounds [5,7,8]. Moreover, oleuropein contains a hydroxytyrosol moiety. Hydroxytyrosol and its derivatives (e.g., oleuropein complex and tyrosol) are the basis of the health claim on olive oil polyphenols approved by the Commission Regulation (EU) No. 432/2012, i.e., “olive oil polyphenols contribute to the protection of blood lipids from oxidative stress” [9]. In addition to the interest that oleuropein may cause, olive leaves extracts can provide a basis for the formulation of functional ingredients since a wide spectrum of bioactivities has been reported [5]. Similarly, olive mill leaves have antioxidant and antibacterial properties [2], but little is known about the oleuropein content.

Moreover, as food additives, olive leaves’ extracts may counteract the loss of oil quality and enhance the stability of edible oils [10].

In this context, obtaining extracts rich in functional plant phytochemicals can be addressed to valorize agroindustrial byproducts [11], but generally the yield of extraction of phenolic compounds is low. This means that there is a large remaining fraction that can be applied for other purposes. Instead of a stand-alone process, the process based on the principles of biorefinery would increase the profitability [12]. This means that olive leafy byproducts can be complementarily used as feedstock for the production of second generation bioethanol from their sugar fraction [4,12]. Another unexplored fraction is proteins. Vegetable proteins can be extracted and used as such or in the form of hydrolyzates, with adequate nutritional and techno-functional properties [13,14,15]. New sources of usable protein could help to alleviate the global feed protein crisis [16]. Alkaline extraction is commonly used to recover plant proteins [13,15], but more studies are required to give new insights into the operational requirements when applied to leafy byproducts.

Therefore, the objective of this study was to integrate the sequential extraction of phenolic compounds, including the valuable compound oleuropein, and proteins from olive mill leaves and olive leaves from field. For that, maceration and ultrasound-assisted extraction of phenolic compounds was performed, while alkaline extraction was optimized via response surface methodology (RSM) to recover proteins and establish crucial factors affecting this step. Moreover, the residues obtained after extraction were characterized in terms of sugars and lignin since it can be valuable for other uses under a biorefinery approach.

## 2. Materials and Methods

### 2.1. Chemicals and Standards

For extraction, ethanol and sodium hydroxide (NaOH) were obtained from Sigma-Aldrich (St. Louis, MO, USA) and VWR Chemicals (Radnor, PA, USA), respectively. Acetone, methanol and acetonitrile were purchased from PanReac AppliChem (Barcelona, Spain). Folin and Ciocalteu’s phenol reagent, sodium carbonate, 2,2′-azinobis(3-ethylbenzothiazoline-6-sulfonate) (ABTS), 6-hydroxy-2,5,7,8-tetramethylchroman-2-carboxylic acid (trolox), and standards of oleuropein, luteolin 7-*O*-glucoside and gallic acid were purchased from Sigma-Aldrich. Ultrapure water was obtained by a Milli-Q system (Millipore, Bedford, MA, USA).

### 2.2. Samples

Olive mill leaves from ‘Picual’ olive trees were collected in 2016 from the olive mill “SCA Unión Oleícola Cambil” (Jaén, Spain). Moreover, olive leaves were picked randomly in 2018 from olive tree leaves (‘Picual’) located in the Campus “Las Lagunillas” (University of Jaén). Leaves were washed with tap water, air-dried, and stored in a dry place until use. Just before starting the extraction process, both samples were ground (particle size around 1 mm) with an Ultra Centrifugal Mill ZM 200, Retsch (Haan, Germany).

### 2.3. Chemical Composition of Leafy Byproducts and Extraction Residues

The moisture and ash contents were determined according to the standard National Renewable Energy Laboratory (NREL) procedure [17]. According to the aforementioned procedure and after acid hydrolysis, carbohydrates were determined by high-performance liquid chromatography (HPLC) and lignin by gravimetric analysis. Acid soluble lignin was determined at 205 nm and a coefficient of extinction of 110 L/g cm was used [18]. The cellulose content was estimated from the glucose using an anhydro correction of 0.90 and hemicellulose from the other sugars using an anhydro correction of 0.90 and 0.88 for hexoses and pentoses, respectively [4,19].

### 2.4. Ethanolic Extraction

Extraction of phenolic compounds was based on the procedure of Ammar et al. [20], with some modifications. Briefly, olive leafy samples were extracted at 1:20 of solid-to-liquid ratio of initial weight using ethanol. Each sample was placed in a test tube, sonicated (40 kHz) (Ultrasons, J.P. Selecta, Barcelona, Spain) for 30 min at room temperature and centrifuged at 4000 rpm (Herolab, Wiesloch, Germany) for 15 min. Then, the supernatants were collected. For analysis, samples were filtered with a syringe filter (nylon, 0.45 μm pore size) (SinerLab Group, Madrid, Spain) and stored at 20 °C until analysis. Moreover, a portion of the extracts (15 mL) were oven-dried at 40 °C until constant weight. A control without sonication was also performed.

### 2.5. Alkaline Extraction

Alkaline extraction was initially performed at pH 9 in a bath (JULABO GmbH, Seelbach, Germany) at 60 °C and under agitation during 125 min. For that, NaOH at 0.03 M was added to olive mill leaves at a solid-to-liquid ratio of 1:10. These conditions were selected to be in the range of those reported in literature [13].

Then, the extraction conditions were optimized by RSM and the protein recovery was evaluated. The effect of NaOH concentration, extraction time and temperature were tested at three experimental levels using a central composite design (CCD) (2^3^ + star, face centered). A total of 18 assays were carried out in randomized run order: eight points of a full factorial design (combination of levels 1 and −1), six star points, and four center points to estimate the experimental error. The assays were firstly performed at: i) mild conditions: pH 6–9 (i.e., NaOH concentration from 0.008 to 0.1 M); time, 10–240 min; temperature, 40–80 °C, and then ii) using strong conditions: NaOH concentration, 0.1–0.7 M; time, 10–240 min; temperature, 60–100 °C. The goodness of fit of the model was evaluated by the coefficient of determination (*R*^2^), the lack of fit, and the residual standard deviation (RSD). The extraction at the optimum conditions were applied to olive leaves from mill and field and three repetitions were performed for each type of leaves.

In all cases, after subsequent centrifugation, which was performed at 4000 rpm (Herolab) for 15 min, supernatants were collected and stored at −20 °C until further analysis. Moreover, a portion of the extracts (15 mL) were oven-dried at 40 °C until constant weight.

### 2.6. Total Phenol Content (TPC) Method

The TPC was determined by a colorimetric assay using Folin–Ciocalteu reagent in 96-well polystyrene microplates, according to Mekky et al. [21]. A Bio-Rad iMarkTM microplate absorbance reader (Hercules, CA, USA) was employed. The absorbance was measured after incubation for 2 h in dark and compared with a calibration curve of gallic acid (25 to 300 μg/mL, *R*^2^ > 0.99). The results were expressed as gallic acid equivalents (GAE).

### 2.7. Trolox Equivalent Antioxidant Capacity (TEAC) Assay

The TEAC assay was performed using the aforementioned microplate reader and following the procedure described by [21]. ABTS radical was produced by reacting ABTS with 2.45 mM potassium persulfate. The mixture was kept in dark at room temperature for 24 h and then diluted with water till reaching an absorbance value of 0.70 (± 0.02) at 734 nm. Afterwards, this solution and the extract (appropriately diluted) were mixed in the proportion 10:1 (*v/v*) and the absorbance measured. Absorbance readings were compared to a standard calibration curve of trolox (6 to 330 μM, *R*^2^ > 0.99) and the results expressed as trolox equivalents (TE). Moreover, caffeic acid was used as a positive control (TEAC value ≈ 1.4 ± 0.1 mmol TE/mmol of compound).

### 2.8. Reversed-Phase (RP)-HPLC Analyses

The ethanolic extracts were analyzed using RP-HPLC coupled to UV. For that, a Shimadzu Prominence UFLC device was used, which was equipped with a DGU-20A5 degasser, LC-20AD quaternary pump, SIL-20AC HT auto sampler, SPD-M20A diode array detector and CTO-10AS VP column oven. A BDS HYPERSIL C18 column (290 mm × 4.6 mm, 5 μm particle size, Thermo Fisher Scientific Inc., Waltham, USA) was applied to separate the phenolic compounds. The mobile phase consisted of ultrapure water/0.2% orthophosphoric acid (solvent A), methanol (solvent B), and acetonitrile (solvent C) with an initial composition of 96/2/2 (*v/v/v*). A gradient elution at a flow rate of 1.0 mL/min and 30 °C was performed according to [22]. The obtained extracts were directly injected (20 μL) and the detection was performed in the UV range from 190 to 350 nm. Finally, for quantification, calibration curves at 280 nm were prepared with standards (from 2.5 to 1000 mg/L). The curves (*R*^2^ > 0.99) were *y* = 30,405*x* − 113,090 for luteolin 7-*O*-glucoside and *y* = 5591*x* + 11,911 for oleuropein.

Additionally, RP-HPLC-MS and –MS^2^ (working in automatic mode) was used to confirm the identity of the compounds. This analysis was performed on an Agilent 1100 HPLC System (Agilent Technologies, Waldbron, Germany) connected on-line to an Esquire 6000 ion trap (Bruker, Bremen, Germany). A linear gradient of solvent B (acetonitrile with formic acid, 0.1%, *v/v*) in A (water with formic acid, 0.1%, *v/v*) at a flow rate of 0.5 mL/min was applied according to Ammar et al. [20]. The column was a C18 Kinetex (2.1 × 50 mm, 2.7 µm) (Phenomenex, Barcelona, Spain) and the injection volume was 10 µL. Spectra were recorded over the mass-to-charge (*m/z*) range of 100–1200 in the negative ionization mode. Auto MS/MS analyses were performed at 0.6 *V*. About 4 spectra were averaged in the MS analyses and about 2 spectra in the MS/MS analyses. The data were processed using DataAnalysis (version 4.0) from Bruker.

### 2.9. Protein Content

The crude protein content of the byproducts was determined from the nitrogen content obtained by elemental analysis (TruSpec Micro, Leco, St. Joseph, MI, USA), applying a conversion factor of 6.25. The determination of the soluble protein was based on the Bradford assay, using a commercial kit from Bio-Rad. The absorbance was measured at 595 nm using the aforementioned colorimeter and bovine serum albumin (BSA) was used as standard for quantification to build a calibration curve up to 740 µg/mL (*R*^2^ > 0.99). The protein recovery (%) was estimated as the ratio of protein content in the supernatant to the protein content of the byproducts.

### 2.10. Sodium Dodecyl Sulphate (SDS)-Polyacrylamide Gel Electrophoresis (PAGE)

For SDS–PAGE analysis, 100 µL of protein extract were precipitated by adding 400 µL of acetone for 20 min at cold conditions. The proteins were collected by centrifugation at 10,000× *g* for 10 min at 4 °C, and the resulting pellet was dissolved in 50 µL of Laemmli sample buffer containing 5% (*v/v*) 2-mercaptoethanol, according to [23]. In order to determine the molecular weight of the extracted protein products, their separation was carried out on Mini-PROTEAN^®^ TGX™ Precast Gels (Bio-Rad). Electrophoresis was performed at constant voltage (200 *V*) using Tris/Glycine/SDS buffer (Bio-Rad) as running buffer. Then, gels were stained during 1.5 h with Coomassie Brilliant Blue R-250 staining solution (Bio-Rad). Finally, gels were washed with a solution composed of water/methanol/acetic acid (60%:40%:10%, *v/v*) overnight. The molecular mass markers Precision Plus Protein™ Standard Unstained (10–250 kDa) (Bio-Rad) were used.

### 2.11. Sugar and Sugar Alcohol Analysis

Alkaline extracts were acidified using HCl 2 M (till pH around 3.5) and centrifuged as in Section 2.5. A portion of the supernatants was filtered (nylon, 0.45 μm pore size; SinerLab Group) and analyzed using an ICSep ICE-COREGEL-87H3 column (Transgenomic, Inc., Omaha, NE, USA) according to Martínez-Patiño et al. [24] and other portion was subjected to acid hydrolysis at 120 °C and analyzed as in 2.3.

### 2.12. Statistical Analysis

Statgraphics Centurion (StatPoint Technologies, Inc., Warrenton, VA, USA) was used to build the response surface experimental design and to obtain Pareto charts, which were used to summarize graphically and display the relative importance of each factor. One-way analysis of variance (ANOVA) followed by the least significant difference (LSD) multiple range test at the 0.05 significance level were also performed using the aforementioned software. The data are expressed as mean ± SD (*n* = 3).

## 3. Results and Discussion

### 3.1. Raw Composition of Leafy Byproducts

Table 1 shows the chemical composition of the olive byproducts after conditioning (drying and milling). Differences were found between both leaves types in terms of protein, cellulose (estimated as glucose), hemicellulose, lignin, ash, and mannitol (*p* < 0.05). In this regard, the hemicellulosic sugars of olive leafy biomass are mainly composed of xylose, arabinose, and galactose, which could come from xylans, arabinans, and galactans, respectively [25]. Although, other authors suggest that arabinans and galactans appear to be part of pectins, at least in the initial synthesis [26].

Concerning the nitrogen content in lignin, it could be derived from complexes formed between proteinaceous materials and lignin [27], was similar for both byproducts (*p* = 0.09), but there were differences between the percentage of acid insoluble protein with respect to the total protein content (*p* < 0.05), i.e., above 29% and 16% in olive mill leaves and olive leaves, respectively. Among other factors, all these differences could be explained by its primary origin since olive mill leaves consist mainly of olive leaves but mixed with a small amount of fine wood from small tree branches (<0.5 cm). As commented before, this byproduct is generated during olive harvesting and separated from olives using pneumatic separation systems in the mill.

### 3.2. Evaluation of the Extractions Schemes on Phenolic Compounds, Proteins, Sugars, and Lignin

#### 3.2.1. Evaluation of the Extractions Schemes on Olive Mill Leaves

Preliminarily, three schemes for the sequential extraction of phenolic compounds (maceration or ultrasound-assisted extraction) and proteins (alkaline extraction) from olive mill leaves were evaluated: (Scheme 1) maceration, as control, followed by alkaline extraction; (Scheme 2) alkaline extraction followed by ultrasound-assisted extraction; and (Scheme 3) ultrasound-assisted extraction followed by alkaline extraction. For phenolic extraction, ethanol was selected as solvent since it has several advantages: among others, it is reusable, nontoxic with food grade status [27], as well as a potential biorefinery coproduct. Concerning alkaline extraction, mild conditions (initial pH 9; temperature, 60 °C; time, 125 min) were applied, according to those previously reported [13]. Table 1 shows the values for TPC, the content of the olive bioactives, oleuropein and luteolin 7-*O*-glucoside, and the antioxidant activity determined by TEAC.

**Figure 1:**
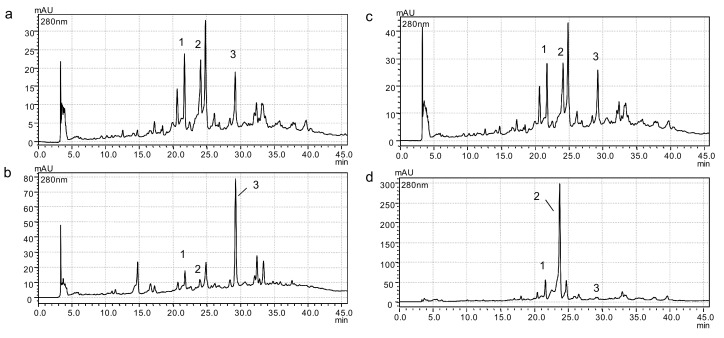
Chromatograms (280 nm) of ethanol extracts obtained by: (**a**) maceration of olive mill leaves (OML) before protein extraction (Scheme 1), (**b**) ultrasound-assisted extraction of OML after protein extraction (Scheme 2), (**c**) ultrasound-assisted extraction of OML before protein extraction (Scheme 3), and (**d**) ultrasound-assisted extraction of olive leaves before protein extraction (Scheme 3). (1) Luteolin 7-*O*-glucoside, (2) oleuropein, and (3) luteolin.

Using ultrasound-assisted extraction (Scheme 3) to recover phenolic compounds and as first step, higher values for TPC, oleuropein content, luteolin 7-*O*-glucoside content and TEAC were obtained as compared to solely maceration (Scheme 1) (Table 2); between 4% and 34% higher. The use of ultrasound generally favors the extraction of phenolic compounds from plant materials, but this enhancement depends on the conditions used (including the device) and the biomass type [21,28]. In another context, when ultrasound-assisted extraction was performed after protein extraction (Scheme 2), the phenolic profile changed mainly quantitatively (Figure 1), and the amounts of oleuropein and luteolin 7-*O*-glucoside were lower (Table 2). Oleuropein could suffer thermal degradation during alkaline extraction following this scheme, caused by oxidation, cleavage of covalent bonds or enhanced oxidation reactions as suggested by [29]. Moreover, the use of alkaline conditions could modify oleuropein to give low active degradation products, as suggested by Soler-Rivas et al. [30]. On the contrary, the values for TPC and TEAC were the highest using this scheme (Scheme 2). This could be also related to the change in the phenolic profile that led to obtain more luteolin in this extract (Figure 1). This fact can explain these results taking into account the results by Benavente-García et al. [7], who reported the antioxidant activity of some olive phenolic compounds, including luteolin, luteolin 7-*O*-glucoside, and oleuropein, using this antioxidant assay. Thus, the phenolic composition and the antioxidant activity of the extracts can be modulated by the sequential extractions scheme applied. Nonetheless, the TEAC method is primarily governed by steric considerations of the radical, and the presence of hydrogen atom transfer-acting antioxidants, which react slowly in this system, could be underestimated [31].

**Figure 2:**
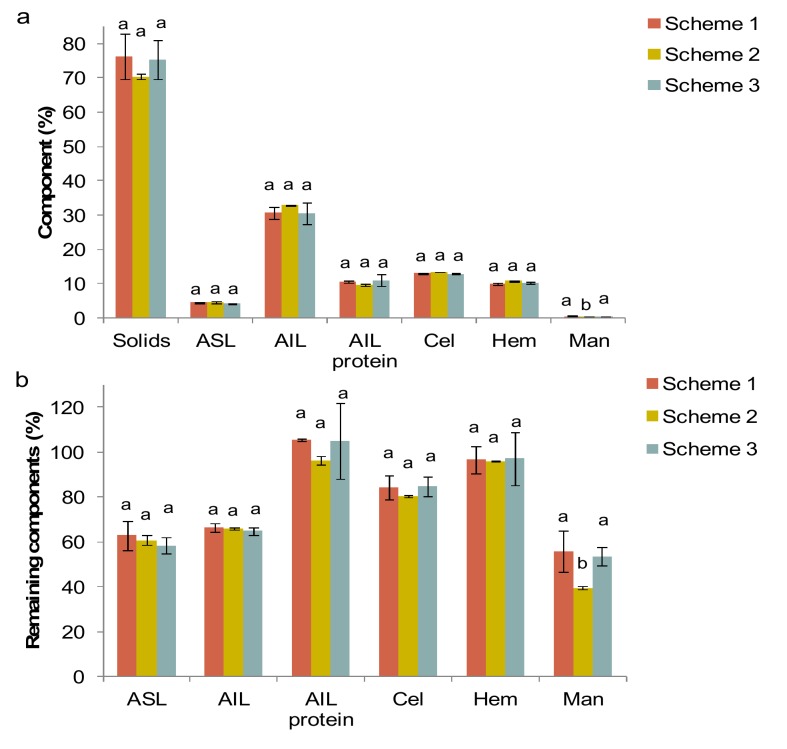
(**a**) Yield of solids (%) and content (%) of acid-soluble lignin (ASL), acid-insoluble lignin (AIL), protein in AIL, cellulose (Cel), hemicellulose (Hem), and mannitol (Man) in the remaining fraction from olive mill leaves (OML) after extraction using Schemes 1–3. (**b**) Corresponding recovery values (%) with respect to the initial amounts in OML.

Concerning the recovery of proteins, there were no differences among the extraction schemes (Table 2). Although the study of Karki et al. [32] suggested that a pretreatment with ultrasound may enhance protein release from soy meal, the byproduct type, the ultrasonic device and the conditions applied were different. Moreover, our results suggested that the residues of extraction contained similar amount of solids, ash, acid-soluble lignin (ASL), acid-insoluble lignin (AIL), protein linked to AIL, cellulose, and hemicellulose (Figure 2). In all cases, the most susceptible fraction was lignin with only 60%–65% remained in the residue after extraction. This makes sense since alkaline pretreatments are used for removing lignin from the biomass in order to increase the accessibility and digestibility of cellulose for saccharification and transformation into biofuels [33].

#### 3.2.2. Comparison between Olive Mill Leaves and Olive Leaves from Field

Overall, since oleuropein is a potential therapeutic molecule as commented before, the extraction of phenolic compounds was performed before alkaline extraction in subsequent experiments since the rest of constituents were not significantly affected by the extractions sequence followed. Nevertheless, if higher antioxidant activity is desired, protein extraction can be performed before phenolic extraction. If the use of ultrasound is not possible in the industrial scheme, the phenolic amounts in the extracts will be reduced only slightly.

Therefore, for comparison, olive leaves were also extracted by ultrasound-assisted extraction as first extraction step (Table 2). This extract presented higher TPC, oleuropein content, luteolin 7-*O*-glucoside content and antioxidant activity than that obtained from olive mill leaves. These differences could be explained by the fact that the composition of the byproducts is different. As commented before, olive mill leaves not only contain leaves but also present woody material. It seems that the content of oleuropein in olive wood is lower than in leaves [20,34,35], being absent in the wood of some cultivars [35]. Also, storage time and conditions could have affected the latter values, since olive leaves were picked fresh before extraction, while olive mill leaves were stored at room temperature as it was at the mill. In any case, olive mill leaves are a cheap and easily accessible source of oleuropein. This means that this leafy byproduct is localized and stored in the mill, being ready for utilization, but storage time and conditions should be further controlled in a future biorefinery based on the production of antioxidant extracts.

### 3.3. Evaluation of the Solubilization of Proteins by Mild Alkaline Conditions after Phenolic Extraction

Alkaline extraction is commonly employed to extract proteins from vegetable sources, but it has not been well explored in leafy byproducts [13,36]. Thus, RSM was applied to evaluate the effect of some parameters on the solubilization of proteins from olive mill leaves using mild alkaline-thermal conditions at a solid-to-liquid ratio of 1:10. Table S1 shows the experimental levels of the tested factors, i.e., initial pH from 6 to 9, extraction time from 10 to 240 min, and temperature from 40 to 80 °C, along with the results obtained for the response variable (protein recovery) and the yield.

**Figure 3:**
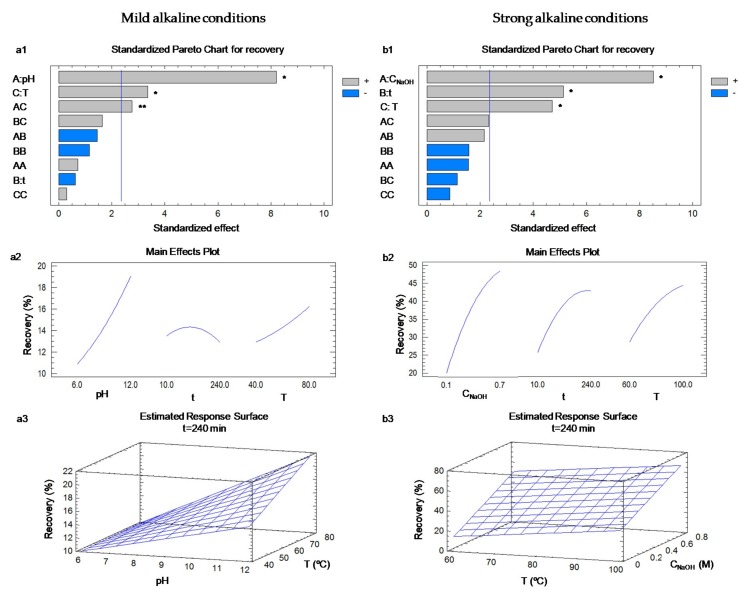
Pareto charts indicating the weight of each factor on the protein recovery and corresponding main effects plots using (**a1** and **a2**, respectively) mild and (**b1** and **b2**, respectively) strong alkaline conditions. The surface plots are represented take into account the significant factors: (**a3**) mild and (**b3**) strong alkaline conditions. C_NaOH_, NaOH concentration; *T*, temperature; *t*, extraction time. * Significant at *p*-value < 0.05; ** significant at *p*-value < 0.1.

Figure 3(a1) shows the Pareto chart of the standardized effect of each term on the protein recovery and its statistical significance at the 90% confidence level, while Figure 3(a2) shows the main effects plots. The Pareto chart (Figure 3(a1)) indicates that pH, temperature, and the interaction of both parameters had the strongest influence on the protein recovery, as well as this effect was positive. The rest of the variables, including the extraction time and the quadratic terms of the parameters, had no significant effects on the extraction recovery, hence they were eliminated from the model. In this way, the corresponding surface plot is depicted in Figure 3(a3). The model had an *R*^2^ of 85.16%, the standard error of the estimate was 1.83 and the *p*-value of the lack-of-fit was 0.82 (Table 3).

The model proposed was:

(1) $$Y=a_{0}+\;\overset{3}{\underset{i=1}{\sum }}a_{i}\;X_{i}+\overset{3}{\underset{i=1}{\sum }}a_{ii}\;X_{i}^{2}+\overset{3}{\underset{i\neq j=1}{\sum }}a_{ij}\;X_{i}\;X_{j}$$

where *Y* is the response variable, a_0_ is a constant, a_i_, a_ii_, and a_ij_ are the linear, quadratic, and interaction coefficients, respectively. The values of the coefficients are shown in Table 3. Using this model, the optimum conditions were: pH 12 (NaOH concentration of 0.1 M), 80 °C and 240 min. The predicted recovery value was 21.5%, which was similar to the experimental value (21.7% ± 2.3%). Moreover, these extraction conditions were applied to olive leaves, but the recovery of proteins was slightly lower at 15.5% ± 0.2%. In this regard, protein extractability depends on the byproduct type and composition [13,37]. Olive leaves contain more cellulose than olive mill leaves (Table 1) and cellulose may hamper the extractability of proteins [37], explaining at least in part our results.

### 3.4. Evaluation of the Solubilization of Proteins by Strong Alkaline-thermal Conditions

**Figure 4:**
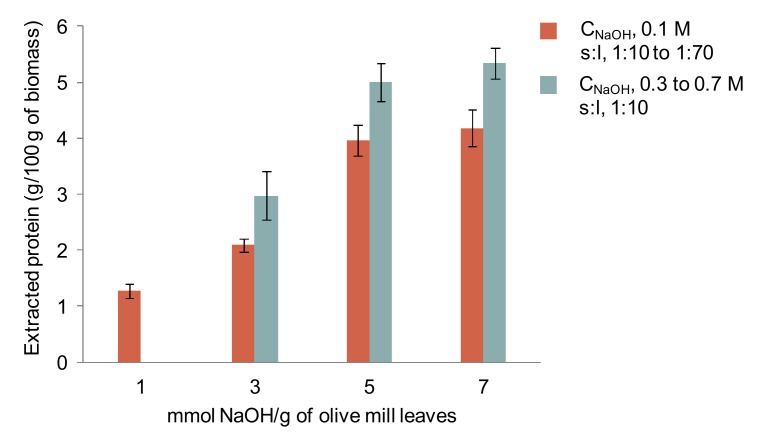
Amount of protein extracted using different ratio of alkali to solid, which was obtained using different NaOH concentration (C_NaOH_) and solid-to-liquid ratio (s:l) values.

Since temperature and pH (determined by the NaOH amount) were the most important factors in the former design, a new design was built to evaluate the effect of stronger NaOH concentration and temperature. Firstly, the effect of the amount of alkali added per solid (1–7 mmol NaOH/g of solid) on the solubilization of proteins was evaluated. For that, NaOH solutions from 0.1 to 0.7 M at a fixed solid-to-liquid ratio of 1:10 were tested, and as well a fixed value of NaOH 0.1 M at different solid-to-liquid ratio values (1:10–1:70). Figure 4 depicts that the use of higher amounts of alkali increased the amount of protein extracted, particularly when using concentrated NaOH solutions. This led to higher pH values (up to 13.3) than using the other way (up to 12.6). These results agree with those obtained by Zhang et al. [38], who reported that the amount of applied alkali is critical to extract proteins from leafy byproducts.

Secondly, taking into account the previous results, the solid-to-liquid ratio was fixed again to 1:10 to reduce the consumption of water and the NaOH concentration (0.1–0.7 M), the temperature (60–100 °C) and the extraction time (10–240 min) were optimized by using a CCD (Table 4). In this case, NaOH concentration, temperature and extraction time had the strongest influence on the protein recovery (*p*-value < 0.05), as well as this effect was positive (Figure 3(b1)). In this case, the interaction and quadratic terms had no significant effects on the protein recovery. The influence of these three operational parameters has also been reported in algae [39] and tea byproduct [38]. Furthermore, the use of high temperatures seems to be essential to extract proteins from leafy byproducts in agreement with Sari et al. [37].

Finally, the model was rebuilt considering only the significant variables and the surface plot is shown in Figure 3(b3). The new model explained almost 70% of the variability and the standard error of the estimate was 5.3%. Since the *p*-value for lack-of-fit in the ANOVA was greater than 0.05 (Table 3), the model appears to be adequate for the observed data at the 95.0% confidence level. Table 3 also details the coefficients for Equation (1) and the optimum conditions, which were obtained using NaOH 0.7 M at 100 °C for 240 min. The predicted recovery value was 62.7%, which is similar to the experimental value (63.1% ± 5.7%), i.e., ≈ 5 g/100 g of olive mill leaves. Finally, the optimum conditions were applied to olive leaves. The recovery value was 55.5% ± 4.3% (i.e., ≈ 5 g/100 g of OL); again it was slightly lower than that for olive mill leaves.

It should be noticed that a recovery value higher than 50% was obtained using the conditions assayed in the experiment 16 (NaOH 0.4 M, 80 °C for 240 min) (Table 4). Although this value is lower than that using the optimum conditions, the alkali and temperature requirements are lesser. This treatment was also applied in the subsequent experiments.

### 3.5. Characterization of the Protein Products by SDS-PAGE

The solubilized protein consisted of proteins partially hydrolyzed into peptides with molecular weight lower than 10 kDa (band B1), proteins/peptides closer to 10 kDa (band B2) and 100 kDa (band B3) (Figure S1). When using stronger thermal-alkaline conditions, bands B1 and B2 were more prominent, suggesting it may favor protein hydrolysis, in agreement with Fetzer et al. [14]. Nonetheless, Zhang et al. [40] reported that tea protein was not severely hydrolyzed after alkaline treatment at 95 °C, 0.1 M NaOH and a *v/w* of 40:1. Moreover, the band B4 (>250 kDa) was not well resolved and could be possibly formed by complexed proteins too large to enter the gel [15].

Some similar bands have been previously reported in olive leaves [22,41], while RuBisCO main subunit band at 55 kDa was not detected. This protein could be affected by hydrolysis reactions occurred under the conditions applied or complexation. Furthermore, all these protein bands were also observed in the alkaline extract from olive leaves, suggesting that the protein precursors are similar for both byproducts (Figure S2).

### 3.6. Characterization of the Residual Fraction after the Sequential Extraction of Phenolic Compounds and Protein

**Figure 5:**
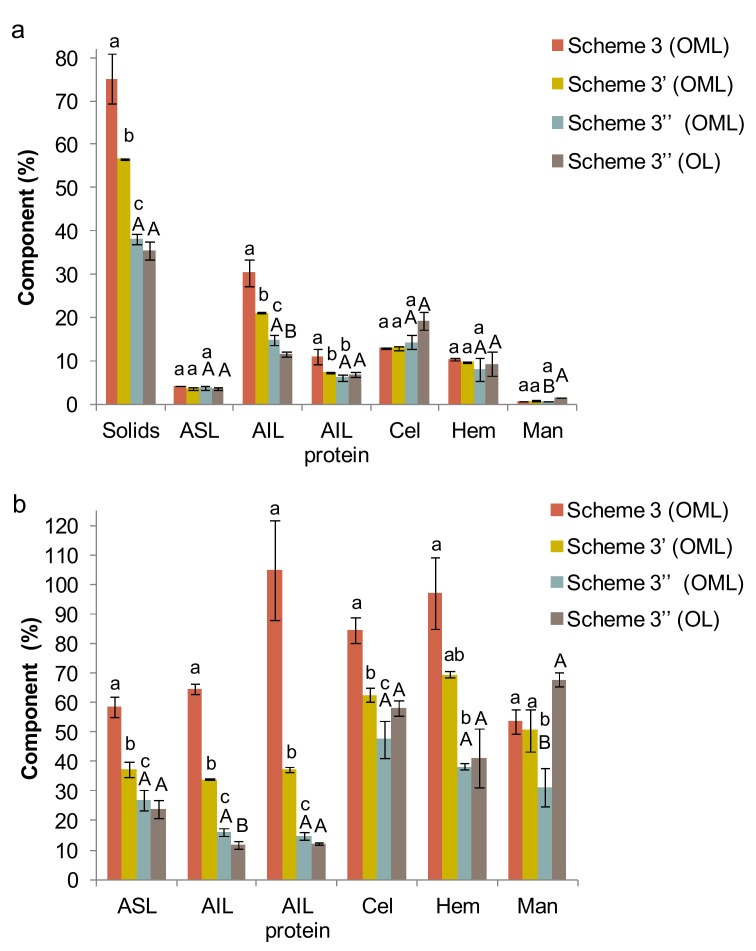
(**a**) Yield of solids (%) and content (%) of acid-soluble lignin (ASL), acid-insoluble lignin (AIL), protein in AIL, cellulose (Cel) and hemicellulose (Hem) and mannitol (Man) in the remaining fraction from olive mill leaves (OML) and olive leaves (OL) after phenolic extraction followed by alkaline extraction using NaOH 0.03 M, 60 °C, 125 min (Scheme 3), NaOH 0.4 M, 80 °C, 4 h (Scheme 3′), and NaOH 0.7 M, 100 °C, 4 h (Scheme 3′′, optimum conditions). (**b**) Corresponding recovery values (%) with respect to the initial amounts in both byproducts.

For a complete valorization of olive mill leaves, the remaining fraction obtained after the sequential extractions scheme was characterized (Figure 5). Under optimum alkaline extraction conditions (i.e., NaOH concentration, 0.7 M; temperature, 100 °C; time, 4 h) (Scheme 3′′), the percentage of lignin was lower than using softer thermal-alkaline conditions, i.e., Scheme 3 (NaOH concentration, 0.03 M; temperature, 60 °C; time, 125 min) and scheme 3′ (NaOH concentration, 0.4 M; temperature, 80 °C; time, 4 h) (Figure 5a). This means that the chemical profiles are different from each other and with respect to the raw byproduct. Particularly, using Scheme 3′′, the recovery of most components was lower, which could pass to the liquid phase as hydrolyzed forms (Figure 5b). Similarly, the latter extraction conditions also changed the chemical profile of the remaining fraction recovered from olive leaves compared to the raw byproduct. In this case, the chemical profile and the recovery values were similar to those of olive mill leaves, with the exception of AIL and mannitol.

In general, the lowest recovery value was found for lignin (both soluble and insoluble fractions) and AIL protein (or protein linked to lignin) (Scheme 3′′), suggesting that these components were more solubilized than the others. It seems that an alkali treatment can attack mainly hydrolysable linkages in lignin, which cause a reduction in the degree of polymerization and disruption of the lignin structure of biomass [42]. This can lead to the breakage of lignin linkages, such as aryl–ether, ester and C–C bonds [43], as well as linkages between lignin–carbohydrate complexes [44], whose presence has been reported in different types of biomass [45]. Among the first type, β-*O*-4′ alkyl–aryl ethers are the most abundant lignin inter-unit linkages in olive tree pruning, which is composed of olive leaves, thin branches and wood [46]. Nonetheless, these authors suggested that its low syringyl/guaiacyl ratio and the presence of condensed structures (C–C) make it probably less reactive than other biomasses, and thus requiring higher amounts of alkali. Furthermore, the destabilization of the polymeric structure of the biomass could favor the release proteins linked to fibers, but literature information is scarce. In our case, the alkaline extraction also led to obtain cellulose and hemicellulose enriched fractions with higher ratios of sugars/lignin, which can be further valorized for obtaining biofuels as shown McIntosh and Vancov [47] for alkaline pretreated wheat straw.

Alternatively, the solubilization of lignin and its co-precipitation with proteins via acid precipitation (until ≈pH 3.5) can explain, at least in part, that the protein enrichment was modest as suggested other authors [13,48]. The protein content of acid precipitates was up to 24% (Scheme 3′). In this context, future studies should be addressed to separate proteins from solubilized lignin and sugars in the alkaline extracts since all these components are valuable, e.g., using enzymes and acids [48]. Concerning sugars and derivatives, mannitol (1.0–2.3 g/L), xylitol (0.4–1.1 g/L), and arabinose oligomers (21.5–35.8 g/L) were detected in the alkaline extracts. The formers have many applications as natural sweeteners and excipients in the food industry and pharmaceutical industries [4], while the latter could be useful as a prebiotics [49].

## 4. Conclusions

The following scheme could be applied to obtain phenolic compounds and proteins from olive leafy byproducts: ultrasound-assisted extraction of phenolic compounds to recover oleuropein followed by alkaline extraction of proteins. The amount of oleuropein extracted per 100 g of biomass was higher in olive leaves (≈1.4 g) than in olive mill leaves (≈0.2 g), while the extracted protein (≈5 g) amount was similar. If higher antioxidant activity is desired, protein extraction can be performed before phenolic extraction, also increasing the luteolin content. Furthermore, to increase the recovery of proteins from this leafy byproduct, strong alkaline-thermal conditions are required. Alkaline extraction led to changes in the residual lignocellulosic fraction, which was enriched in cellulose. Further studies are required to assess its viability for obtaining biofuels in biorefinery and to purify proteins and other interesting compounds, such as oligosaccharides.

## Figures and Tables

**Table 1:** Chemical composition of olive mill leaves (OML) and olive leaves (OL) ^1^.

Component (%)	OML	OL
Crude protein	8.10 ± 0.38 ^1^	9.34 ± 0.35 ^1^
Cellulose (as glucose)	11.73 ± 0.14 ^1^	15.84 ± 0.29 ^1^
Hemicellulose	7.92 ± 0.05 ^1,2^	8.62 ± 0.10 ^1,2^
Mannitol	0.76 ± 0.02 ^1^	2.81 ± 0.03 ^1^
Acid soluble lignin	5.40 ± 0.09 ^1^	7.46 ± 0.37 ^1^
Acid insoluble lignin	35.16 ± 0.05 ^1,3^	28.85 ± 1.05 ^1,3^
N in acid-insoluble lignin	1.06 ± 0.11 ^1^	0.82 ± 0.04 ^1^
Ash	10.04 ± 0.08 ^1^	6.24 ± 0.06 ^1^

^1^ Dry basis. ^2^ Composed of arabinose, xylose, and galactose. ^3^ With N.

**Table 2:** Total phenolic yield (%), total phenol content (TPC) (mg gallic acid equivalents/100 g), oleuropein (Ole) content (mg/100 g), luteolin 7-*O*-glucoside (L7G) content (mg/100 g) and antioxidant activity (µmol trolox equivalents/100 g) of the ethanolic extracts as well as protein recovery (%) after alkaline extraction obtained by three different sequential extractions schemes.

Byproduct ^#^	Scheme ^‡^	Phenolic Yield	TPC/Biomass	TPC/Extract	Ole/Biomass	Ole/Extract	L7G/Biomass	L7G/Extract	TEAC ^#^/Biomass	TEAC ^#^/Extract	Protein Recovery
OML	1	10.1 ± 0.2 ^b^	454 ± 36 ^b^	4476 ± 284 ^a^	142 ± 3 ^ab^	1395 ± 6 ^ab^	31 ± 4 ^ab^	309 ± 46 ^a^	2024 ± 2 ^b^	18,234 ± 139 ^b^	11.2 ± 0.8 ^a^
OML	2	11.7 ± 0.2 ^a^	719 ± 89 ^a^	6167 ± 847 ^a^	79 ± 0.3 ^b^	675 ± 6 ^b^	28 ± 0.3 ^b^	237 ± 6 ^b^	3087 ± 143 ^a^	25,459 ± 2809 ^a^	13.7 ± 1.4 ^a^
OML	3	11.7 ± 0.6 ^aA^	585 ± 24 ^abB^	4998 ± 65 ^aB^	191 ± 45 ^aB^	1790 ± 434 ^aB^	38 ± 3 ^aB^	338 ± 21 ^aB^	2193 ± 69 ^bB^	19,050 ± 101 ^bB^	12.5 ± 0.8 ^a^
OL	3	10.8 ± 0.8 ^A^	1405 ± 99 ^A^	13,108 ± 1877 ^A^	1365 ± 6 ^A^	12,694 ± 694 ^A^	97 ± 1 ^A^	903 ± 8 ^A^	6364 ± 38 ^A^	59,651 ± 6429 ^A^	ND

^#^ OML, olive mill leaves; OL, olive leaves; TEAC, trolox equivalent antioxidant capacity. ^‡^ Sequential extractions. Scheme 1: maceration with ethanol before protein extraction; Scheme 2: ethanolic extraction assisted by ultrasound after protein extraction; Scheme 3: ethanolic extraction assisted by ultrasound before protein extraction. In each column, data followed by the same minor letter are not statistically different from each other concerning the three schemes applied on OML, while data followed by the same capital letter are not statistically different from each other for the Scheme 3 applied in OML and OL (least significant difference test, *p* < 0.05).

**Table 3:** Model equation coefficients and optimum conditions values for the recovery of proteins from olive mill leaves obtained using mild and strong alkaline extractions.

Equation Terms	Mild Alkaline Conditions	Strong Alkaline Conditions
Coefficients ^1^		
a_0_	10.785	−27.166
Linear		
a_1_	−0.174 ***	47.100 ***
a_2_	NS	0.074 **
a_3_	−0.147 **	0.392 **
Interaction		
a_12_	NS	NS
a_13_	0.026 *	NS
a_23_	NS	NS
Quadratic		
a_11_	NS	NS
a_22_	NS	NS
a_33_	NS	NS
*R* ^2^	0.85	0.69
Lack-of-fit	0.823	0.113
Optimum (estimated)	22.3	62.7
Optimum (experimental)	21.7 ± 2.3	63.1 ± 5.7
1 (pH/C_NaOH_)	12	0.7 M
2 (time)	240 min	240 min
3 (temperature)	80 °C	100 °C

^1^ The factors were pH (1), time (2) and temperature (3) in the design for mild alkaline conditions and NaOH concentration (C_NaOH_) (1), time (2) and temperature (3) in the design for strong alkaline conditions. NS, not significant; significant at *** *p* < 0.01; ** 0.01 < *p* < 0.05; * 0.05 < *p* < 0.1.

**Table 4:** Protein recovery from olive mill leaves subjected to different alkaline-thermal treatments (strong conditions).

Assay No.	pH	NaOH Concentration (M)	Time (min)	Temperature (°C)	Protein Recovery (%)	Yield (Solids) (%)
1	13.2	0.4	125	80	36.1	40.0
2	13.6	0.7	240	60	43.4	57.4
3	13.5	0.7	240	100	55.8	66.5
4	12.2	0.1	240	100	15.5	24.4
5	12.3	0.1	240	60	14.4	23.4
6	12.2	0.1	10	100	13.6	23.2
7	13.3	0.4	10	80	13.6	32.1
8	13.4	0.4	125	80	38.4	42.2
9	13.1	0.7	10	60	17.1	50.3
10	13.7	0.7	125	80	51.3	60.1
11	12.5	0.1	10	60	10.0	39.2
12	12.7	0.1	125	80	16.7	21.2
13	12.7	0.4	125	100	53.5	50.0
14	13.2	0.7	10	100	43.9	63.3
15	13.3	0.4	125	80	36.8	42.6
16	13.4	0.4	240	80	54.3	49.3
17	12.8	0.4	125	60	19.0	41.6
18	13.2	0.4	125	80	50.9	46.1

## Supplementary Materials

The following are available online at https://www.mdpi.com/2304-8158/8/11/531/s1, Table S1: Protein recovery from olive mill leaves subjected to different alkaline-thermal treatments (mild conditions). Figure S1: SDS-PAGE of protein extracts from olive mill leaves obtained at different extraction conditions, Figure S2: SDS-PAGE of protein extracts from olive mill leaves (OML) and olive leaves (OL) obtained using 0.7 M NaOH at 100 °C for 4 h.
