# Supplementary material for: Integrated Process for Sequential Extraction of Bioactive Phenolic Compounds and Proteins from Mill and Field Olive Leaves and Effects on the Lignocellulosic Profile
Source: Foods. 2019 Oct 29;8(11):531. doi: 10.3390/foods8110531 (PMC6915506; doi:10.3390/foods8110531)
Supplement: Supplementary file 1 [file foods-08-00531-s001.pdf]

Article

Integrated process for sequential extraction of bioactive phenolic compounds and proteins from mill and field olive leaves and effects on the lignocellulosic profile

María del Mar Contreras [1,*], Antonio Lama-Muñoz [1], José Manuel Gutiérrez-Pérez [1,2], Francisco Espínola [1,2], Manuel Moya [1,2], Inmaculada Romero [1,2] and Eulogio Castro [1,2]

[1] Department of Chemical, Environmental and Materials Engineering, University of Jaén, Campus Las Lagunillas, 23071 Jaén, Spain; mcgamez@ujaen.es (M.d.M.C.); alama@ujaen.es (A.L.-M.); jmgp0014@red.ujaen.es (J.M.G.-P.); fespino@ujaen.es (F.E.); mmoya@ujaen.es (M.M.); iromero@ujaen.es (I.R.); ecastro@ujaen.es (E.C.)

[2] Center for Advanced Studies in Energy and Environment, University of Jaén, Campus Las Lagunillas, 23071 Jaén, Spain.

* Correspondence: mcgamez@ujaen.es; mmcontreras@ugr.es; mar.contreras.gamez@gmail.com; Tel.: +34-953-21-27-99 (M.d.M.C.)

Supplementary material

Tables and figures

Table S1. Protein recovery from olive mill leaves subjected to different alkaline-thermal treatments (mild conditions).

Assay nº	pH	NaOH concentration (M)	Time (min)	Temperature (ºC)	Protein recovery (%)	Yield (solids) (%)
1	9	0.03	125	60	12.3	16.3
2	12	0.1	240	40	11.4	13.0
3	6	0.008	240	80	10.8	14.8
4	12	0.1	240	80	21.7	22.6
5	6	0.008	10	80	10.3	15.1
6	9	0.03	125	60	14.8	18.6
7	9	0.03	125	60	15.9	18.4
8	12	0.1	10	40	17.8	20.4
9	12	0.1	125	60	20.4	21.5
10	9	0.03	10	60	12.7	15.6
11	9	0.03	240	60	14.1	16.9
12	6	0.008	125	60	10.0	15.0
13	12	0.1	10	80	20.8	21.6
14	9	0.03	125	60	13.4	17.0
15	6	0.008	10	40	9.8	13.5
16	9	0.03	125	80	16.0	17.8
17	6	0.008	240	40	10.3	14.6
18	9	0.03	125	40	13.6	16.8

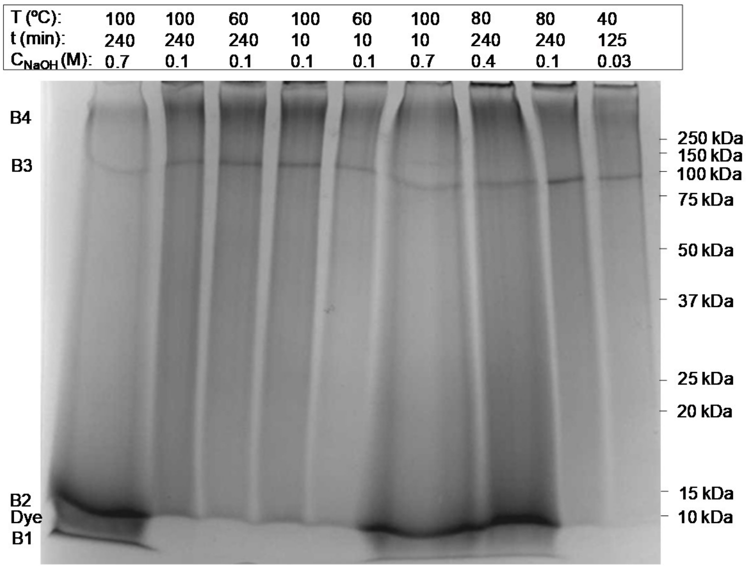

Figure S1. SDS-PAGE of protein extracts from olive mill leaves obtained at different extraction conditions.

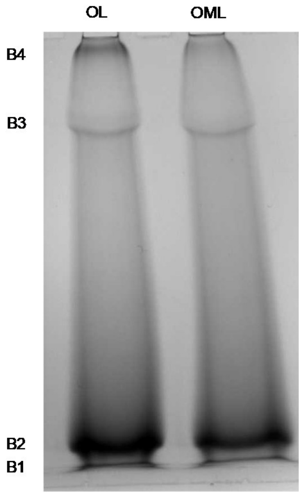

Figure S2. SDS-PAGE of protein extracts from olive mill leaves (OML) and olive leaves (OL) obtained using 0.7 M NaOH at 100 ºC for 4 h.
